# Low-Power GaAlAs Laser Irradiation Promotes the Proliferation and Osteogenic Differentiation of Stem Cells via IGF1 and BMP2

**DOI:** 10.1371/journal.pone.0044027

**Published:** 2012-09-04

**Authors:** Jyun-Yi Wu, Yan-Hsiung Wang, Gwo-Jaw Wang, Mei-Ling Ho, Chau-Zen Wang, Ming-Long Yeh, Chia-Hsin Chen

**Affiliations:** 1 Institute of Biomedical Engineering, National Cheng Kung University, Tainan, Taiwan, Republic of China; 2 School of Dentistry, College of Dental Medicine, Kaohsiung Medical University, Kaohsiung, Taiwan, Republic of China; 3 Orthopaedic Research Center, College of Medicine, Kaohsiung Medical University, Kaohsiung, Taiwan, Republic of China; 4 Department of Orthopaedics, College of Medicine, Kaohsiung Medical University, Kaohsiung, Taiwan, Republic of China; 5 Department of Orthopaedics, Kaohsiung Medical University Hospital, Kaohsiung Medical University, Kaohsiung, Taiwan, Republic of China; 6 Department of Orthopaedic Surgery, University of Virginia, Charlottesville, Virginia, United States of America; 7 Department of Physiology, College of Medicine, Kaohsiung Medical University, Kaohsiung, Taiwan, Republic of China; 8 Graduate Institute of Medicine, College of Medicine, Kaohsiung Medical University, Kaohsiung, Taiwan, Republic of China; 9 Department of Physical Medicine and Rehabilitation, Kaohsiung Medical University Hospital, Kaohsiung, Taiwan, Republic of China; 10 Department of Physical Medicine and Rehabilitation, Faculty of Medicine, College of Medicine, Kaohsiung Medical University, Kaohsiung, Taiwan, Republic of China; 11 Department of Physical Medicine and Rehabilitation, Kaohsiung Municipal Ta-Tung Hospital, Kaohsiung, Taiwan, Republic of China; Instituto de Engenharia Biomédica, University of Porto, Portugal

## Abstract

Low-power laser irradiation (LPLI) has been found to induce various biological effects and cellular processes. Also, LPLI has been shown to promote fracture repair. Until now, it has been unclear how LPLI promotes bone formation and fracture healing. The aim of this study was to investigate the potential mechanism of LPLI-mediated enhancement of bone formation using mouse bone marrow mesenchymal stem cells (D1 cells). D1 cells were irradiated daily with a gallium-aluminum-arsenide (GaAlAs) laser at dose of 0, 1, 2, or 4 J/cm^2^. The lactate dehydrogenase (LDH) assay showed no cytotoxic effects of LPLI on D1 cells, and instead, LPLI at 4 J/cm^2^ significantly promoted D1 cell proliferation. LPLI also enhanced osteogenic differentiation in a dose-dependent manner and moderately increased expression of osteogenic markers. The neutralization experiments indicated that LPLI regulated insulin-like growth factor 1 (IGF1) and bone morphogenetic protein 2 (BMP2) signaling to promote cell proliferation and/or osteogenic differentiation. In conclusion, our study suggests that LPLI may induce IGF1 expression to promote both the proliferation and osteogenic differentiation of D1 cells, whereas it may induce BMP2 expression primarily to enhance osteogenic differentiation.

## Introduction

In the United States, approximately 1.5 million fractures are attributed to osteoporosis annually, and the expenditure for osteoporotic fractures is calculated to be 13.8 billion dollars [1]. Osteoporosis is a common skeletal disorder that is characterized by reduced bone mineral density (BMD) and disrupted bone microarchitecture, which can cause bone fragility and increase the risk of bone fracture [2]. The risk factors for osteoporosis include aging, nutrition (vitamin D deficiency and excess alcohol), medication (steroid use), and life style factors (physical activities that decrease bone-loading) [3]. Currently, the major treatment for osteoporosis is hormonal therapy (estrogen supplements), bisphosphonates, calcium and vitamin D supplements, and exercise [4].

Mesenchymal stem cells (MSCs) can be harvested from many tissues, such as bone marrow, the umbilical cord, liver, and adipose tissue. The mouse bone mesenchymal stem cell (BMSC) line, D1, was derived from bone marrow. This cell line has been characterized as multipotent, with osteogenic, chondrogenic, and adipogenic potential. D1 cells exhibit a primarily osteogenic phenotype, and they have been used in fracture repair and osteointegration of prosthetic implants [5], [6]. MSC-based bone tissue engineering also represents a new approach for the treatment of osteoporosis and osteoporosis-mediated fractures.

Low-power laser irradiation (LPLI) consists of non-thermal irradiation at wavelengths between visible light and the near-infrared range. LPLI has been reported to have medical benefits in wound healing [7], [8], pain relief [9], reduction of inflammation [10], and promotion of microvascularization and angiogenesis [11]. The irradiated cells absorb the light, triggering intracellular signaling cascades that can lead to various biological effects, including cell growth, proliferation, collagen synthesis, and differentiation. These biological effects have been reported in several cell types, such as endothelial cells [11], fibroblasts [12], and MSCs [13], [14]. Recently, some reports have indicated that LPLI therapy may facilitate new bone formation. Additionally, LPLI may have a beneficial effect on bone fracture repair by increasing alkaline phosphatase (ALP) levels [15], bone density [16], and bone matrix formation [17]. Based on in vitro studies, LPLI may enhance the viability of osteoblasts [18], [19] as well as the osteogenic biostimulatory effect on osteoblast-like cells [20]. However, the precise molecular mechanisms of LPLI-mediated biostimulatory effects remain unclear.

In this study, we investigated the effects and the molecular mechanisms of a LPLI on the proliferation and osteogenic differentiation of D1 cells.

**Figure 1:**
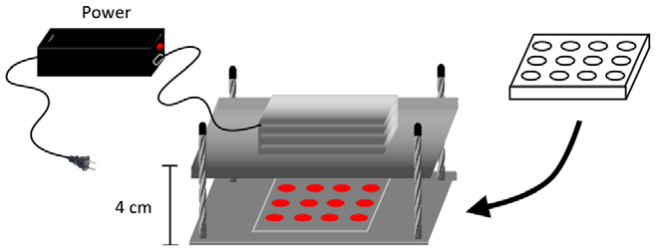
Schematic diagram of the laser apparatus, the mechanism can produce 12 laser beams at the same time. Cell culture plates were put on the base of the machine and the pillars were adjusted to match the target area of irradiation.

**Table 1:** Primer sequences used for Real-Time PCR.

Gene	Primer	Accession No.
BMP2	F: 5′ – AGC TGC AAG AGA CAC CCT TTG – 3′	NM_007553
	R: 5′ – AGC ATG CCT TAG GGA TTT TGG A – 3′	
OC	F: 5′ – GAG GGC AAT AAG GTA GTG AAC A – 3′	BC165978
	R: 5′ – AAG CCA TAC TGG TCT GAT AGC TCG – 3′	
ALP	F: 5′ – AAC CCA GAC AGC ATT CC – 3′	AB473959
	R: 5′ – GTC AGT CAG GTT GTT CCG ATT CAA – 3′	
RUNX2	F: 5′ – CCC AGC CAC CTT TAC CTA CA – 3′	NM_001145920
	R: 5′ – TAT GGA GTG CTG CTG GTC TG – 3′	
RANKL	F: 5′ – CAC CAT CAG CTG AAG ATA GT – 3′	AB036798
	R: 5′ – CCA AGA TCT CTA ACA TGA CG – 3′	
OPG	F: 5′ – ACC ACT AGC TCC CAA GGT TCC – 3′	U94331
	R: 5′ – ACA GCC ACT TGT TCA TTG TGG TC – 3′	
IGF1	F: 5′ – GCA TTG TGG ATG AGT GTT GC – 3′	NM_001111276
	R: 5′ – TCC TTT GCA GCT TCG TTT TC – 3′	
β-actin	F: 5′ – CCA ACC GTG AAA AGA TGA CC – 3′	XR_030256
	R: 5′ – ACC AGA GGC ATA CAG GGA CA – 3′	

## Materials and Methods

### Cell Culture

D1 cells, which are multipotent MSCs cloned from the bone marrow of BALB/c mice [21], were purchased from the American Type Culture Collection (ATCC) and maintained in bone medium (BM) containing Dulbecco’s Modified Eagle Medium (DMEM, GIBCO), 10% fetal bovine serum (FBS, Biosciences), 50 mg/ml sodium ascorbate (Sigma), non-essential amino acids and 100 U/ml penicillin/streptomycin (GIBCO) in a humidified 5% CO_2_ atmosphere at 37°C. For osteogenic differentiation, cells were grown to 80% confluence and changed to osteo-induction medium (OIM). OIM contained 10^−7^ M dexamethasone (Sigma), 50 µM L-ascorbate-2-phosphate (Sigma), and 10 mM β-glycerophosphate disodium (Sigma) as described by Wang et al. [22].

**Figure 2:**
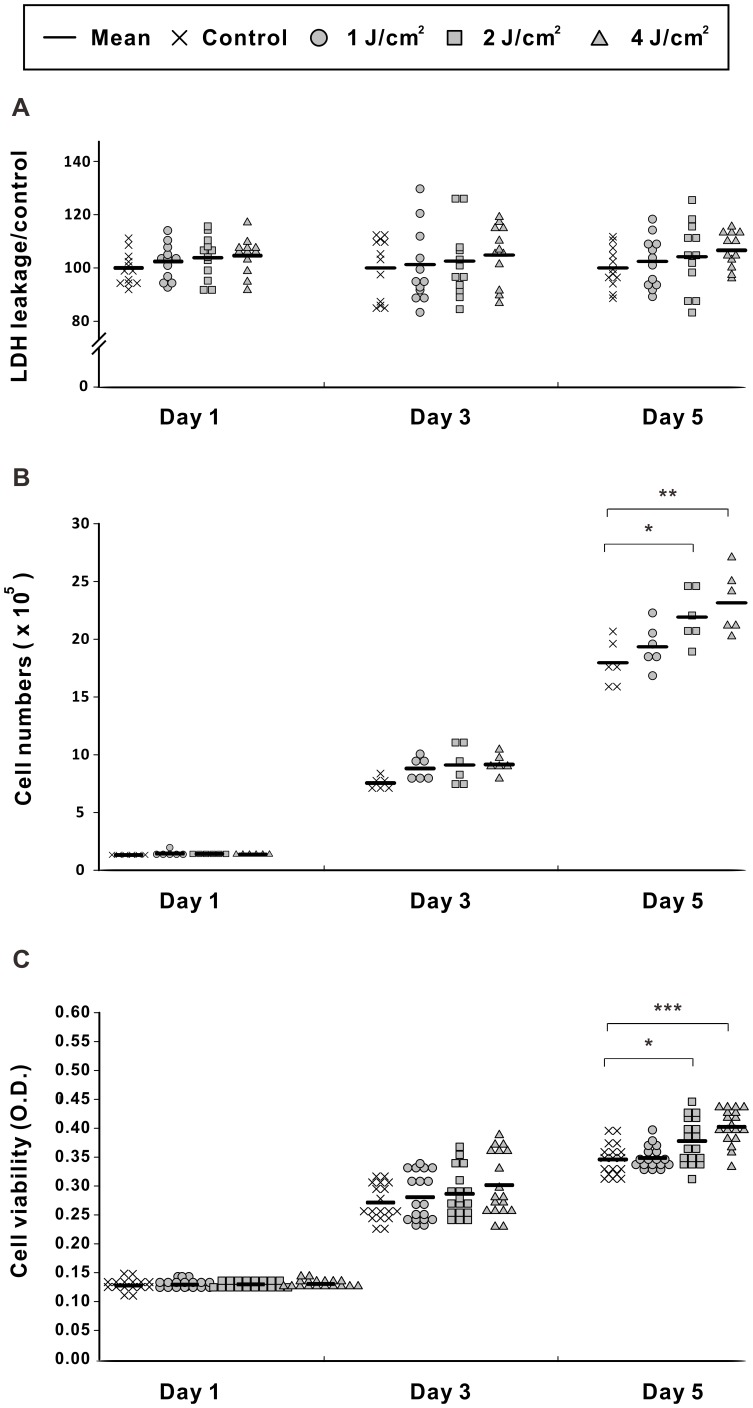
LPLI enhances the proliferation of D1 cells. Cells were treated with LPLI at doses of 0 (control), 1, 2, or 4 J/cm^2^ and assayed at days 1, 3 and 5. (**A**) LDH leakage was analyzed to evaluate cell cytotoxicity. There were no significant differences between the groups (n = 12). (**B**) Cell numbers were counted at indicated times. There were no differences between the cell numbers of each group at day 1 and day 3. At day 5, the cell numbers of the LPLI groups treated with 2 and 4 J/cm^2^ were significantly higher than that of control group (n = 6). (**C**) An MTT assay was performed, and the optical densities were measured. Similar results with the cell counting analysis was found (n = 18). The statistical significance levels were as follows: * p<0.05, ** p<0.01, and *** p<0.001 compared with the control group.

**Figure 3:**
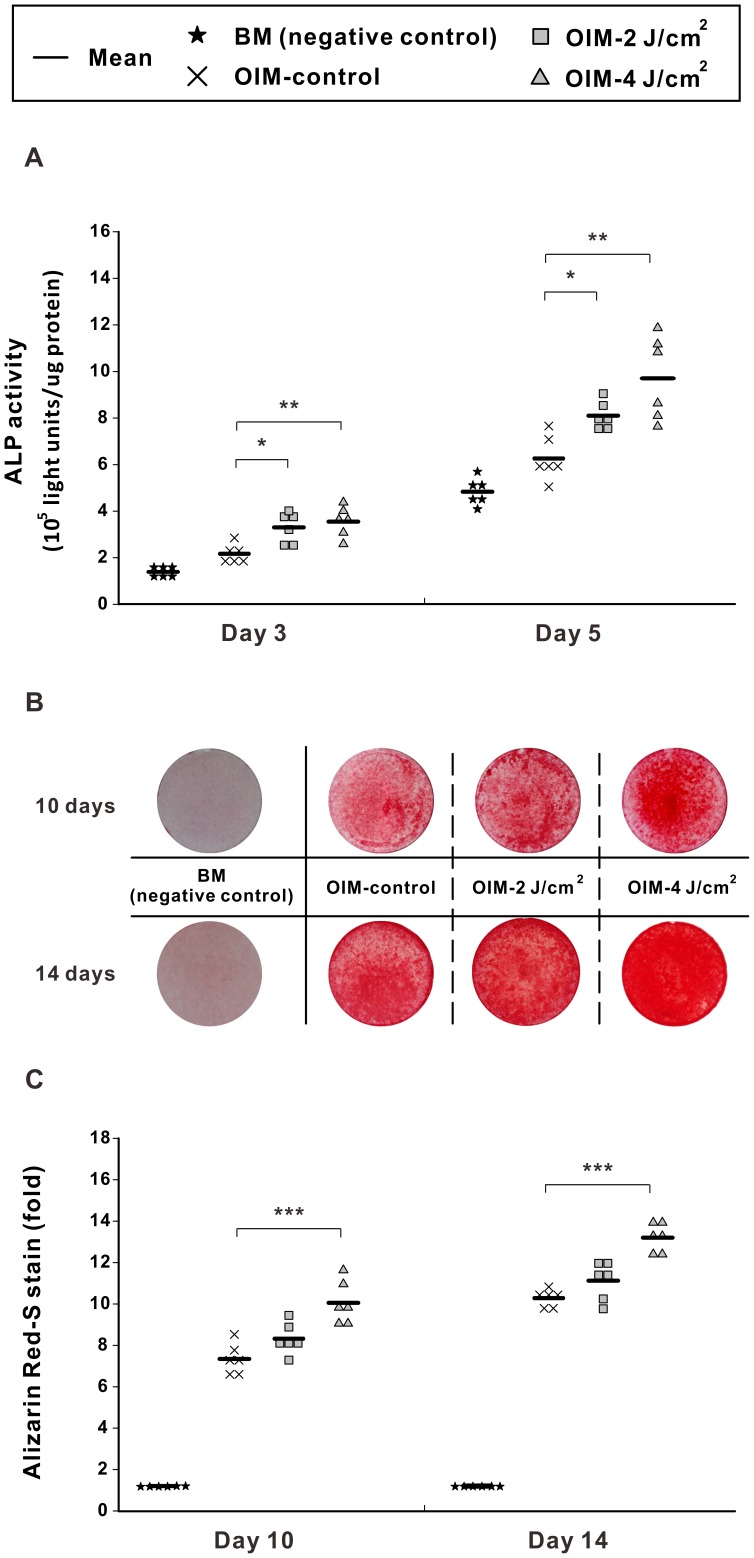
LPLI promotes osteogenesis in D1 cells. D1 cells were cultured in BM (negative control) or OIM (OIM-control), or D1 cells were cultured in OIM and treated with LPLI at 2 J/cm^2^ (OIM-2 J/cm^2^) or 4 J/cm^2^ (OIM-4 J/cm^2^). (**A**) The ALP activity of D1 cells was analyzed. LPLI at both 2 J/cm^2^ and 4 J/cm^2^ significantly increased the ALP activity at days 3 and 5 (n = 6). (**B**) D1 cells were cultured for 10 or 14 days. Following fixation, the cells were stained with Alizarin Red S. A pronounced dose-dependent increase of mineralization was observed at both day 10 and day 14 (n = 6). (**C**) Relative quantification of the Alizarin Red S staining shown in B (n = 6). The following statistical significance levels were applied: * p<0.05, ** p<0.01, and *** p<0.001 compared with the OIM-control group.

**Figure 4:**
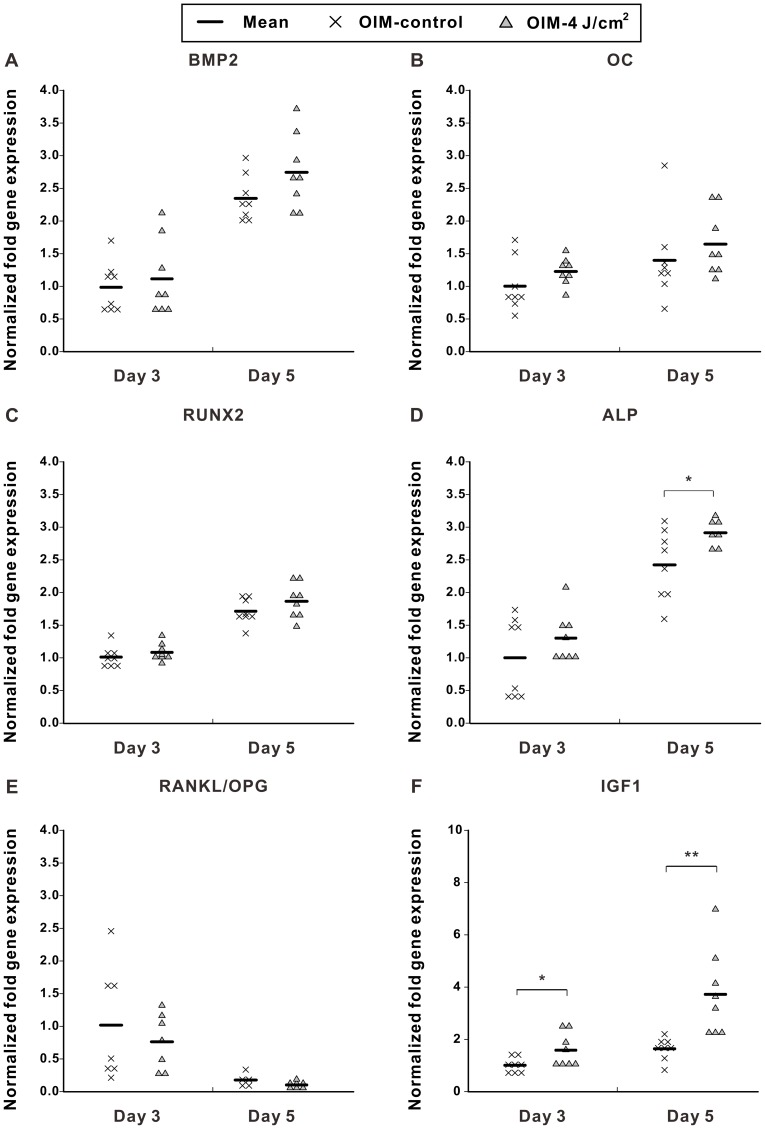
LPLI regulates different osteogenic differentiation markers. (**A**) *BMP2.* (**B**) *OC.* (**C**) *RUNX2.* (**D**) *ALP.* (**E**) *RANKL*/*OPG.* (**F**) *IGF1*. LPLI moderately increased osteogenic gene (*BMP2*, *OC*, *RUNX2*, and *ALP*) expression and decreased osteoclastogenic gene (RANKL/OPG) expression. The results were analyzed by the 2^−ΔCT^ method based on the OIM-control at day 3 (n = 8). The following statistical significance levels were applied: *p<0.05 and **p<0.01, compared with OIM-control.

**Figure 5:**
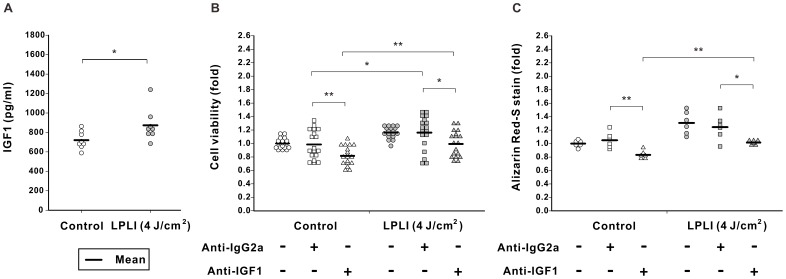
IGF1 regulated LPLI-mediated proliferation and osteogenic differentiation of D1 cells. (**A**) D1 cells were treated with LPLI at doses of 0 (control) or 4 J/cm^2^. IGF1 protein levels in conditioned culture medium on day 5 were determined by ELISA. LPLI significantly increased the IGF1 protein levels in culture medium (n = 8). (**B**) D1 cells were irradiated with or without LPLI (4 J/cm^2^) and cultured with an IGF1 neutralizing antibody (anti-IGF1, 10 µg/ml) or the same amount of a control antibody (anti-IgG2a, 10 µg/ml). After culture for 5 days, cell viability was determined by an MTT assay. The proliferation of D1 cells was significantly decreased in the control and the LPLI-stimulated cells by blocking with IGF1 antibody (n = 18). (**C**) Cells underwent a similar treatment as described in B, except that D1 cells were cultured in OIM for 10 days. The relative osteogenic activity was quantified using Alizarin Red S staining. The mineralization was significantly inhibited by treatment with anti-IGF1 antibodies in the control and the LPLI-stimulated cells (n = 6). The following statistical levels were applied: * p<0.05 and ** p<0.01.

**Figure 6:**
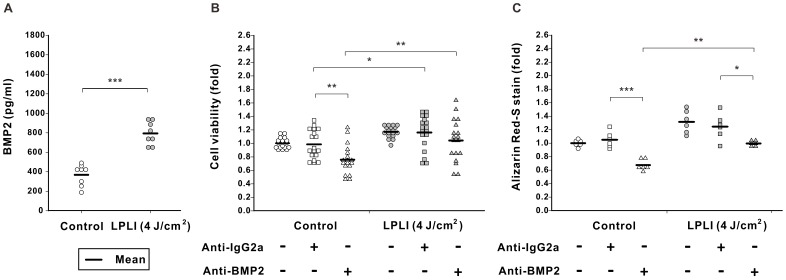
BMP2 regulates LPLI-mediated osteogenic differentiation, but not cellular proliferation. (**A**) D1 cells were treated with LPLI at doses of 0 (control) or 4 J/cm^2^. BMP2 protein levels in conditioned culture medium on day 5 were determined by ELISA. LPLI significantly increased the BMP2 protein levels in culture medium (n = 8). (**B**) D1 cells were irradiated with or without LPLI (4 J/cm^2^) and cultured with a BMP2 neutralizing antibody (anti-BMP2, 10 µg/ml) or the same amount of a control antibody (anti-IgG2a, 10 µg/ml). After culture for 5 days, cell viability was determined by an MTT assay. The proliferative activity was significantly decreased in the control cells, but not in the LPLI-stimulated cells (n = 18). (**C**) Cells underwent a similar treatment as described in B, except that D1 cells were cultured in OIM for 10 days. The relative osteogenic activity was quantified using Alizarin Red S staining. Anti-BMP2 neutralizing antibodies significantly decreased mineral deposition in the control and the LPLI-stimulated cells (n = 6). The following statistical levels were applied: * p<0.05, ** p<0.01 and *** p<0.001.

D1 cells were detached using 1× trypsin (GIBCO) and then seeded onto specific culture plates. The cell density, type of culture plate, laser energy, and irradiation duration used in each experiment are listed in supporting information (Table S1).

### Laser Irradiation

A gallium-aluminum-arsenide (GaAlAs) red laser (wavelength 660 nm) (TRANSVERSE IND. CO., LTD., Taipei, Taiwan) was used as the light source. The laser had a maximum power of 50 mW, and the distance between the laser source and the bottom of the plate could be adjusted to match the intended target size. In this study, the distance between the laser source and the cells was 4 cm (Fig. 1). Under these conditions, the power decayed to 38 mW, and the power density was 10 mW/cm^2^. Therefore, cells were irradiated for 100 s, 200 s, or 400 s to receive energies of 1 J/cm^2^, 2 J/cm^2^, or 4 J/cm^2^. Cells were irradiated once a day, and all irradiation processes were performed on a clean bench at room temperature. The control group was processed under the same conditions but excluding laser irradiation.

### Lactate Dehydrogenase (LDH) Leakage

LDH leakage was measured to quantify cytotoxicity with a Cytotoxicity Detection kit (Roche, Germany). The supernatants and cells in culture were assayed based on the manufacturer’s guidelines. Absorbance was measured by an ELISA reader (Model no. 680, Bio-Rad) at 490 nm. The LDH leakage was calculated by determining the ratio of the absorbance of the supernatant to the absorbance of the supernatant and cell lysate combined.

### Cell Viability

Cell viability was determined by counting cell numbers and using an MTT assay (3-[4,5- dimethylthiazol-2-yl]-2,5-diphenyltetrazolium bromide; Sigma). Briefly, cells were stained with trypan blue (Invitrogen) and counted using a hemocytometer under a microscope (Nikon, Eclipse, TS100). The MTT assay was performed as described previously [23]. The reactions were measured using an ELISA reader at 595 nm, where absorbance is directly proportional to the numbers of viable cells.

### ALP Activity

D1 cells were harvested and rinsed twice with 1x phosphate-buffered saline (PBS; Bio-Tech). Cells were lysed in lysis buffer (0.2% Triton X-100 dissolved in 1x dilution buffer; Tropix, Applied Biosystems) and subjected to sonication. The cell lysate was mixed with equal volumes of reaction buffer and chemiluminescent substance (Applied Biosystems) for 20 min and measured by TopCount in chemiluminescent mode (Packard). The protein concentration was determined using a BCA Protein Assay kit (Novagen) according to the manufacturer's instructions. The ALP activity was normalized to the protein concentration of each sample.

### Alizarin Red S Staining

Cells were washed twice with 1x PBS and fixed in 10% formaldehyde (BS Chemicals) for 10 minutes. The cells were then rinsed twice with deionized water and stained with Alizarin Red S (Sigma) for 10 minutes at room temperature. After staining, the excess dye was removed by washing gently with running water. The calcification deposits typically stained red. Dye was extracted from the stained cells for quantification using 200 µl of 10% glacial acetic acid at 60°C for one hour. The amount of Alizarin Red extracted was determined using an ELISA reader at 490 nm.

### Real-time Reverse Transcription-polymerase Chain Reaction (RT-PCR)

Cells were harvested and rinsed twice with PBS. Total RNA was extracted by adding Trizol reagent (Gibco BRL, Bethesda, MD), separating with chloroform (J.T. Backer), and precipitating with isopropanol (J.T. Backer). The RNA pellet was washed once with 75% ethanol and once with 100% ethanol for dehydration. The RNA was dissolved in diethylpyrocarbonate-treated water, and the concentration of RNA was quantified by measuring the absorbance with a spectrophotometer (ND-1000, NanoDrop) at 260 nm and 280 nm. The first strand of the cDNA was reverse-transcribed by adding 1 µg of RNA, Moloney murine leukemia virus reverse transcriptase, and oligo-dT primers. Quantitative real-time PCR was performed using a Bio-Rad iQ5 real-time detection machine (Bio-Rad Laboratories Inc., Hercules, CA). The reactions were performed in a 12.5 µl mixture containing cDNA, forward and reverse primers for each gene, and SYBR® Green Real-time PCR Master Mix (TOYOBO). The bone morphogenetic protein 2 (*BMP2*), osteocalcin (*OC*), *ALP*, runt-related transcription factor 2 (*RUNX2*), osteoprotegerin (*OPG*), receptor-activated NF-κB ligand (*RANKL*), and insulin-like growth factor 1 (*IGF1*) genes were evaluated. Primer sequences are listed in Table 1. Relative mRNA expression levels were calculated from the threshold cycle (Ct) value of each PCR product and normalized to a housekeeping gene (β-actin) using the comparative Ct method.

### Enzyme-linked Immunoassay (ELISA)

The supernatants of the cell culture were collected to evaluate IGF1 and BMP2 concentrations using the AssayMax Mouse IGF-1 ELISA kit (ASSAYPRO) and the Mouse BMP-2 ELISA kit (IBL-America), respectively. Both kits were used according to the manufacturer's instructions. Protein levels of IGF1 and BMP2 were quantified at 450 nm using an ELISA plate reader.

### Neutralization of IGF1 and BMP2

Neutralizing antibodies against IGF1 and BMP2 (Peprotech) or control IgG2a antibodies (Millipore) were dissolved in deionized water to a final concentration of 1 mg/ml and stored at −20°C. Cells were cultured with the indicated neutralization antibodies or control IgG2a antibodies at 10 µg/ml. The medium containing the antibodies was replaced every two days.

### Statistical Analysis

SPSS version 17.0 was used for the statistical analysis. The results are expressed as the mean ± standard deviation. Statistically significant differences between the control and laser groups were determined using the student *t* test and analysis of variance (ANOVA) followed by a *post hoc* Tukey’s test for multiple comparisons. A *p*-value less than 0.05 was considered statistically significant.

## Results

### LPLI Enhances Cell Proliferation of D1 MSCs

The cytotoxic effects of LPLI on D1 cells were measured by analyzing the leakage of LDH. The LDH values were normalized to the LDH values of the control group, and the results showed that LDH leakage increased slightly with increasing LPLI energy at each time point (Fig 2A). However, the LPLI treatments did not induce cytotoxic effects on D1 cells, even when the cells were exposed to the highest dose.

After irradiation, the number of D1 cells was slightly higher than the control at day 1 and day 3, but these differences were not statistically significant (Fig. 2B). Nevertheless, cell counts remained higher in the LPLI group at day 5 and the cell count following 2 and 4 J/cm^2^ of irradiation was significantly higher than that of the control group (*p*<0.05 for 2 J/cm^2^, p<0.001 for 4 J/cm^2^) (Fig. 2B). Similar effects on cell viability were observed with the MTT assay (Fig 2C). The optical densities in the MTT assay increased with time at all energy levels of LPLI. Compared to the control group, the optical densities of laser-irradiated cells were higher at day 1 and day 3, but no significant differences were found. At day 5, the MTT activities were significantly higher in cells treated with 4 J/cm^2^ (*p*<0.001) and 2 J/cm^2^ LPLI (*p*<0.05) than in the control group.

### LPLI Enhances Osteogenic Differentiation of D1 Cells

We evaluated the effects of LPLI on osteogenic differentiation of D1 cells using ALP activity and Alizarin Red S staining. Compared to cells cultured in BM (negative controls), cells in OIM displayed increased ALP activity and obvious mineralization (Fig. 3A and 3B). Interestingly, LPLI at both 2 J/cm^2^ and 4 J/cm^2^ significantly enhanced the ALP activity of D1 cells cultured in OIM at days 3 and 5 (Fig. 3A). Furthermore, based on the observation of calcification deposits stained with Alizarin Red S, laser irradiation promoted osteo-differentiation in a dose-dependent manner after culturing the cells in OIM for 10 or 14 days (Fig. 3B). Quantitative determination of Alizarin Red S staining is shown in Fig. 3C. Compared to the non-irradiated OIM control cells, OIM-treated cells that received 2 J/cm^2^ LPLI or 4 J/cm^2^ LPLI showed staining intensity that was greater by 1.13-fold and 1.37-fold on day 10 and 1.08-fold and 1.28-fold on day 14. The OIM and 4 J/cm^2^ LPLI treated group showed significant difference with non-irradiated OIM control group.

### LPLI Regulates Osteogenic Gene Expression

After 3 or 5 days of culture, gene expression of *BMP2*, *OC*, *ALP*, and *RUNX2* was slightly increased when D1 cells were treated with LPLI (4 J/cm^2^) (Fig. 4A-D); however, only the increase in *ALP* expression at day 5 in cells irradiated with 4 J/cm^2^ LPLI was statistically significant (Fig. 4D). Following LPLI, *OPG* expression was increased, but *RANKL* expression was slightly decreased (data not shown). LPLI decreased the *RANKL*/*OPG* ratio but no statistical difference was observed (Fig. 4E). Taken together, LPLI not only modestly increased osteogenic gene expression but also possibly affected osteoclast formation by decreasing the *RANKL*/*OPG* ratio on D1 cells.

### The Biophysiological Effects of LPLI Though BMP2 and IGFI Signaling Pathways

Several growth factors, including platelet-derived growth factor alpha (PDGFα), Transforming growth factor beta (TGFβ), BMP2 and IGF1, have been reported to simultaneously regulate stem cell proliferation and differentiation [23], [24], [25], [26], [27], [28]. To evaluate the possible factors that were involved in the physiological effect of LPLI, we analyzed the mRNA expression of these growth factors by real-time RT-PCR. After culturing for 3 and 5 days, LPLI induced the highest gene expression of *IGF1* (Fig. 4F) when compared with *PDGFα* or *TGFβ* (data not shown).

We further confirmed the protein production of IGF1 and BMP2 in LPLI-stimulated D1 cells. Consistently, ELISA analyses revealed that LPLI significantly increased the IGF1 and BMP2 protein levels in culture medium on day 5 (Fig. 5A and 6A).

We next neutralized IGF1 and BMP2 using specific antibodies to verify which growth factor was important in LPLI-induced cell proliferation and differentiation. After blocking IGF1, the proliferation of D1 cells was significantly decreased in the control and the LPLI-stimulated cells (Fig. 5B). Likewise, osteogenic differentiation was significantly inhibited by treatment with anti-IGF1 antibodies (Fig. 5C). However, the anti-IgG2a treatment did not affect cell proliferation or differentiation.

When D1 cells were treated with anti-BMP2 neutralizing antibodies, the proliferative activity was significantly decreased in the control cells, but not in the LPLI-stimulated cells (Fig. 6B). In the case of LPLI-induced osteogenic differentiation, anti-BMP2 neutralizing antibodies significantly decreased mineral deposition (Fig. 6C). Together, these results indicate that IGF1 is important in LPLI-induced stem cell proliferation and osteogenic differentiation, whereas BMP2 mainly regulates LPLI-induced osteogenic differentiation.

## Discussion

The results of the present study showed that irradiation with a low-power 660 nm GaAlAs red laser at various energy levels affected the cell physiological and molecular properties of D1 cells. The frequency of the laser used in irradiation can also affect cell proliferation. In 2001, Coombe et al. [29] used a GaAlAs laser at a wavelength of 830 nm to treat osteosarcoma cells with a single dose or daily irradiation doses of 0.5, 1, 2, or 5 J. They found no significant difference in cell count or MTT activity between the laser-irradiated and control groups over a period of 10 days. In our preliminary assay, BMSCs were laser-irradiated in the same way. However, our data on cell proliferation showed that the daily irradiation group exhibited significantly higher proliferative activity than the single-irradiation group (data not shown). Therefore, daily irradiation with LPLI was applied in this study.

No apparent cytotoxic effect was observed from the LPLI parameters used in this study, which is consistent with other studies. Mvula et al. [30] found no morphological differences between adipose-derived stem cells that were treated with 5 J/cm^2^ LPLI (635 nm) and those that were not irradiated. Hou et al. [14] also reported that BMSCs showed a small but insignificant increase in LDH leakage under LPLI (635 nm) treatment at energy densities of 0.5, 1, 2, and 5 J/cm^2^. These results indicate that cells can grow effectively during laser irradiation in vitro.

The optimal energy density for stimulating cell proliferation is still under debate. In 1998, Ozawa et al. [31] indicated that significant cell proliferation occurred at the early stages of culture (approximately six days after laser irradiation). After estimating the effect on proliferation at days 1, 3, and 5, they found that LPLI significantly enhanced cell proliferation on day 5. Our results revealed a dose-dependent increase in D1 cell proliferation, with an optimal dose of 4 J/cm^2^. To verify whether higher exposure to LPLI promoted greater cell proliferation, we increased the energy densities to 6 J/cm^2^ and 8 J/cm^2^ and performed an MTT assay. At both of these doses, the MTT activity was reduced (data not shown). Therefore, an energy density of LPLI at 4 J/cm^2^ was a suitable dose for our studies of D1 cell proliferation. Similar to our results, several studies have reported on the optimal conditions for proliferation, including the use of a 660 nm diode laser at an energy density of 4.8 J/cm^2^ on synovial fibroblasts [12], a 660 nm InGaAlP laser at a dose of 3 J/cm^2^ on human dental pulp stem cells [32], a 660 nm diode laser at an energy density of 1.9 J/cm^2^ on BMSCs [33], a 650 nm diode laser at a dose of 2.28 J/cm^2^ on calvarial cells [34], and a 830 nm GaAlAs diode laser at a dose of 3 J/cm^2^ on osteoblastic cells [19]. Together, these results reinforce the observation that LPLI promotes the proliferation of different cell types. However, optimal LPLI treatment conditions for cell proliferation should be further studied with distinct cell types and laser parameters.

ALP is a biomarker that is used to evaluate bone metabolism; staining methods are also commonly used to visualize bone nodule formation. Fukuhara et al. [35] observed that irradiation with a GaAlAs laser (905 nm) at an energy density of 3.75 J/cm^2^ significantly increased bone nodule area, as measured by von Kossa staining and subsequent determination of the number of ALP-positive colonies of calvarial cells. Khrdra et al. [36] showed that ALP activity increased after exposure to 3 J/cm^2^ GaAlAs irradiation (830 nm). Similar results were also observed in our study when we assessed ALP activity and Alizarin Red S staining. A remarkable dose-dependent enhancement of osteogenic induction was observed at both 10 and 14 days. However, based on our quantification of Alizarin Red S staining, the increase at 14 days was not as striking as the induction at 10 days; we attribute this effect to the saturation of osteogenic induction due to the limited cell growth area.

Findings on osteogenic gene expression after LPLI treatment are controversial. Bouvet-Gerbettaz et al. [37] irradiated murine bone marrow cells with a GaAlAr laser (808 nm) at 4 J/cm^2^ and observed no differences in the expression of *BMP2*, *OC*, *ALP*, *RUNX2*, collagen type 1 (*COL1*), or bone sialoprotein 2 (*BSP2*) by quantitative real-time RT-PCR. Nevertheless, several previous studies have reported that LPLI increases the expression levels of osteoglycin (*OGN*) [38], *RUNX2* [35], *ALP*, osteopontin (*OP*), and bone sialoprotein (*BSP*) [39]. In this study, the expression of the osteogenic markers, *BMP2*, *OC*, *ALP* and *RUNX2*, was not consistently increased by LPLI. However, LPLI significantly increased the expression of *ALP* at day 5. To initiate osteoclastogenesis, *RANKL* is expressed by osteoblasts and binds to receptor-activated NF-κB (*RANK*), a membrane-bound receptor in osteoclasts, to induce the maturation of osteoclasts [40]. *OPG* competitively binds to *RANKL* and blocks *RANKL*/*RANK* signaling; therefore, *OPG*, *RANKL*, and *RANK* form a regulatory system to control the bone resorption process [40]. The ratio of the expression of *OPG* and *RANKL* is important in determining bone mass and skeletal integrity. Our results revealed that LPLI increased *OPG* expression but slightly decreased *RANKL* expression and the *RANKL*/*OPG* ratio on D1 cells. Similar results have also been reported by Xu et al. [34]. Even though our data did not show LPLI remarkably decreased *RANKL*/*OPG* ratio, it indicated that LPLI might negatively regulate the activation of osteoclasts. However, the role of LPLI in osteoclastogenesis should be further studied using osteoblast/osteoclast cocultures or animal models.

The mechanisms of LPLI promotion of proliferation and differentiation of cells are still not fully understood. Several growth factors, such as PDGFα [28], TGFβ [26], [27], IGF1 [24], [25], [26], and BMP2 [23], [24], have been reported to regulate stem cell proliferation and differentiation. Our results indicated that LPLI induced higher gene expression of *IGF1* than *PDGFα* and *TGFβ*. Furthermore, both the anti-IGF1 and anti-BMP2 antibodies were used in our study to verify which growth factor was important in LPLI-induced cell proliferation and differentiation. We found that treatment with these two antibodies reduced osteogenic differentiation, but only IGF1 neutralizing antibodies could reduce LPLI-induced cell proliferation. It is an interesting phenomenon that LPLI can regulate different growth factors under different cellular physiological conditions. After LPLI at 4 J/cm^2^, the inhibitory effects of the antibodies on osteogenic differentiation were significantly decreased but were still higher than the inhibitory effects observed in cells to which no antibodies were added. The data indicate that LPLI may regulate proliferation and osteogenic differentiation via IGF1 and BMP2 signaling pathways, but other factors may also be involved. Further studies are necessary to understand the precise signaling pathways involved.

Tissue engineering is a very promising approach for the repair of damaged tissues and organs. The ability of stem cells to proliferate and differentiate plays a critical role in their clinical application. LPLI is an economical and non-contact-based method that can be used to manipulate the activity of cells. Recently, several studies examined the use of LPLI in several animal models and found beneficial effects on tissue healing and regeneration [15], [16], [41]. Here, our results indicate a potential mechanism underlying the LPLI-mediated effects on stem cells and suggest a clinical application for LPLI in stem cell therapy and bone fracture healing.

In conclusion, our results reveal that low power GaAlAs laser irradiation at 4 J/cm^2^ not only had no cytotoxic effects on BMSCs but also promoted their proliferation. A dose-dependent enhancement of osteogenic differentiation by LPLI was found by evaluating ALP activity and mineral deposits. LPLI modestly increased osteogenic gene expression. LPLI-mediated stem cell proliferation and osteogenic differentiation may occur though the BMP2 and IGFI signaling pathways. Therefore, our results provided a potential cellular mechanism of LPLI on stem cells in vitro, and further investigation of LPLI in therapeutic application will be necessary to clarify using in vivo experiments.

## Supporting Information

Table S1 **Experimental design.**
(DOC)
