# Supplementary material for: Low-Power GaAlAs Laser Irradiation Promotes the Proliferation and Osteogenic Differentiation of Stem Cells via IGF1 and BMP2
Source: PLoS One. 2012 Sep 4;7(9):e44027. doi: 10.1371/journal.pone.0044027 (PMC3433487; doi:10.1371/journal.pone.0044027)
Supplement: Table S1 — Experimental design. (DOC) [file pone.0044027.s001.doc]

| **Table S1.** Experiment design | | | | |
| --- | --- | --- | --- | --- |
|  | Cell density (cells/well) | Culture plate | Energy density (J/cm^2^) | Duration (Days) |
| *Cytotoxicity* | | | | |
| LDH assay | 4 × 10^3^ | 96 well | 0, 1, 2, 4 | 1, 3, 5 |
| *Proliferation* | | | | |
| Cell counting | 1 × 10^5^ | 12 well | 0, 1, 2, 4 | 1, 3, 5 |
| MTT assay | 4 × 10^3^ | 96 well | 0, 1, 2, 4 | 1, 3, 5 |
| *Osteogenesis* | | | | |
| ALP activity | 5 × 10^4^ | 12 well | 0, 2, 4 | 3, 5 |
| Alizarin Red stain | 5 × 10^4^ | 12 well | 0, 2, 4 | 10, 14 |
| Real-time PCR | 5 × 10^4^ | 12 well | 0, 4 | 3, 5 |
| *Others* | | | | |
| ELISA | 4 × 10^3^ | 96 well | 0, 4 | 5 |
| The irradiation was started after 24 hours from cell seeding, and performed every day. | | | | |
| All the experiments were executed after 24 hours from last irradiation. | | | | |
| All the experiments were repeated at least three times. | | | | |
